# Comparative evaluation of the effectiveness of concentrated growth factor alone and in combination with diode laser application in the treatment of intrabony periodontal defects: A clinical and radiographic split-mouth study

**DOI:** 10.34172/joddd.40781

**Published:** 2024-06-24

**Authors:** Kalaiselvan Dharani, Jaishree Tukaram Kshirsagar, Priyangha Thangavel

**Affiliations:** ^1^Department of Periodontology and Implantology, Adhiparasakthi Dental College and Hospital, Melmaruvathur, Chengalpattu, The Tamilnadu Dr. MGR Medical University, Chennai, India; ^2^Department of Periodontology, Tamilnadu Government Dental College and Hospital, Chennai, The Tamilnadu Dr. MGR Medical University, Chennai, India; ^3^Department of Periodontology, Saveetha Dental College and Hospitals, Saveetha Institute of Medical and Technical Sciences, Saveetha University, Chennai, India

**Keywords:** Guided tissue regeneration, Lasers, Low-level light therapy, Platelet-derived growth factor, Semiconductor

## Abstract

**Background.:**

Applying autologous growth factors and diode laser in periodontal therapy enhances fibroblast-mediated new attachment and osteoblastic differentiation. Hence, this study compared and evaluated the effectiveness of concentrated growth factor (CGF) alone and with diode laser application in managing intrabony periodontal defects.

**Methods.:**

Ten patients with stage III periodontitis were included in this study. All the patients underwent an open flap debridement (OFD) procedure followed by CGF membrane placement in the intrabony defect in site A, whereas, in site B, after OFD, all the patients underwent diode laser irradiation before CGF membrane placement. Plaque and gingival bleeding index (PI & GBI), PPD, and clinical attachment level (CAL) were evaluated at baseline and 3 and 6 months later. Bone fill (BF), BF%, bone crest changes (BCC), and BCC% were assessed radiographically at six months postoperatively.

**Results.:**

Significant reductions in PI and GBI scores, probing pocket depth (PPD), and CAL gain were observed at both sites 3 and 6 months from baseline. A significant reduction in PPD and CAL gain was noted between sites, which were higher in site B than in site A with a mean difference of 0.70±0.05 mm and 1.30±0.18 mm, 0.90±1.89 mm at 3 and 6 months, respectively. Radiographic measurement showed better BF, BF%, BCC, and BCC% at both sites at six months, which were higher at site B than at site A but statistically insignificant.

**Conclusion.:**

The combination of CGF and diode laser application has demonstrated successful and promising results in terms of regeneration, improving the clinical and radiographic parameters.

## Introduction

 Periodontitis is an infectious inflammatory disease of the periodontium characterized by the progressive and irreversible destruction of tooth-supporting structures. When the resorption of alveolar bone exceeds the formation, the equilibrium is disrupted, resulting in the alteration of sound periodontal morphological and functional characteristics. Destruction of connective tissue and bone loss are the hallmarks of periodontal disease that represent the consequences of the spread of periodontitis.^1^

 Prichard, in 1965, stated that periodontitis-induced bone defects could be inconsistent margins, hemiseptae, interproximal craters, intrabony defects, furcation involvement, and combinations of the above.^2^ Periodontal therapy aims to prevent and arrest periodontal disease progression to maintain the outcomes achieved through therapeutics and regenerate the lost periodontal tissues.

 According to the Glossary of Periodontal Terms in 1992, periodontal regeneration is the reconstruction of lost tissues by restoring the architecture and function. Periodontal attachment gain, pocket depth reduction, and an increase in bone level are the aims of periodontal regenerative therapy.^3^ Treatment modalities such as scaling and root planing, soft tissue curettage, open flap debridement (OFD), and regenerative periodontal flap surgeries with bone grafts and barrier membranes achieve the aims of periodontal regeneration to some extent. However, they fail to regulate the progenitor cells involved in regeneration. The migration, adhesion, proliferation, organization, and maturation of progenitor cells of the periodontium to the denuded root enhance periodontal regeneration.

 Autologous growth factors are bioactive polypeptide proteins rich in regenerative stimulus that regulate the actions of progenitor cells, induce tissue regeneration, and enhance faster healing.^4^ Sacco introduced an advanced platelet concentrate in 2006 and named it a concentrated growth factor (CGF). Since it is an organic matrix rich in fibrin, it constitutes various growth factors and biphasic platelets with abundant leucocytes and stem cells.^5^ Different centrifugation speeds, large and denser fibrin matrix, and biphasic platelets in the CGF enhance increased growth factor release than other platelet concentrates. It enhances connective tissue attachment by improving wound stability and provides a scaffold for cellular migration, proliferation, matrix formation, and osteoid production.^6^

 Laser-assisted periodontal surgery is one recent advancement that enhances regeneration by fibroblast-mediated new attachment and osteoblastic differentiation on the root surface. A Diode laser is a soft tissue laser comprised of a solid active medium from a semiconductor crystal of gallium, aluminum, and arsenic. It penetrates deeper and is absorbed by the pigmented tissues.^7^ The detoxification effect of laser enhances fibroblast attachment and bone matrix formation by removing the epithelial lining and granulation tissue and preventing epithelial downgrowth.^8^ It also empowers the bactericidal effect on periodontal pathogens by the disinfecting thermal effect, thereby enhancing the complete removal of bacteria and toxins from the periodontal pocket.^9^

 The present study evaluated the regenerative efficacy of CGFs with and without laser application in managing intrabony periodontal defects.

## Methods

 The Institutional Ethical Review Board of Tamilnadu Government Dental College and Hospital, India, approved the study protocol under the code 04.01.2021 (4/IERB/2021). Ten systemically healthy patients with an age range of 20‒50 years of either gender with bilateral localized stage III Grade B/C periodontitis with clinical probing depth of ≥ 5 mm following phase I therapy and radiographic evidence of vertical bone loss were selected randomly and included in the study. Patients with poor oral hygiene maintenance after phase I therapy, systemic diseases/metabolic disorders, known allergies, smoking habits, history of present pregnancy, and lactation were excluded.

 The bilateral defects were randomized by coin tossing into experimental sites A and B. At site A, the patients were managed by OFD followed by a CGF membrane placement in the intrabony defect. OFD was performed at site B, followed by diode laser application and a CGF membrane placement in the intrabony defect.

###  Pre-surgical procedures

 A clinical periodontal examination was conducted after complete clinical case recording using the University of North Carolina (UNC)-15 and Naber’s probes. Intraoral periapical radiographs were taken with a radiographic grid using extension cone paralleling (XCP) holders. The acrylic stent was customized to measure probing pocket depth (PPD) and clinical attachment level (CAL) to avoid changes in the angulations of probe placement. Phase I therapy was performed, which included oral hygiene instructions, scaling, and root planing using hand and ultrasonic instruments. Adjunctive chemical plaque control in the form of 0.12% chlorhexidine mouthwash twice daily was advised. Patients were recalled and reviewed after four weeks of phase I periodontal therapy to assess oral hygiene status, PPD, CAL, and bleeding on probing.

###  Preparation of concentrated growth factor membrane

**Figure 1:**
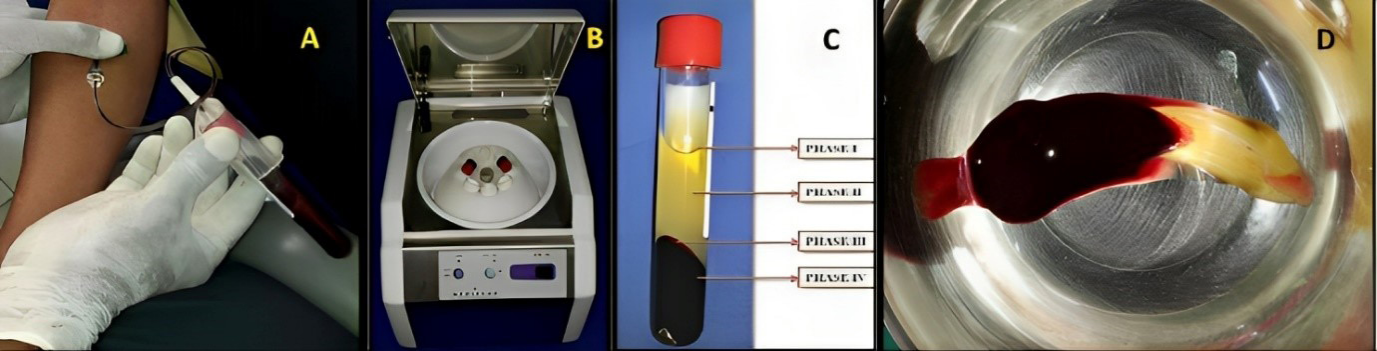
Concentrated growth factor preparation. A. Blood collection. B. Centrifugation in Medifuge. C. Phases of blood fractions after centrifugation. D. Fibrin clot containing CGF. (CGF: concentrated growth factor)

**Figure 2:**
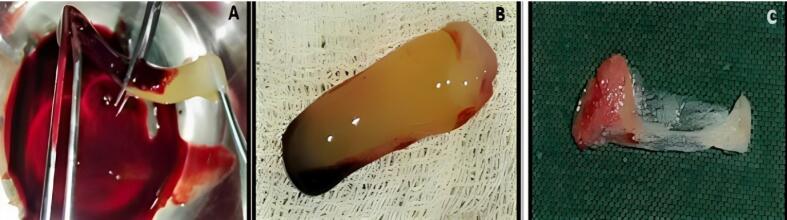
Concentrated growth factor membrane preparation. A. Separation of CGF from RBC layer. B. Separated CGF. C. CGF membrane. (CGF: concentrated growth factor; RBC: red blood cell)

 A total of 10 mL of venous blood was drawn from the patient’s antecubital fossa in sterile test tubes without an anticoagulant solution. The test tubes were immediately centrifuged using a program with the following characteristics: 30 seconds - acceleration; 2700 rotations per minute (rpm) - 2 minutes; 2400 rpm - 4 minutes; 2700 rpm - four minutes; 3000 rpm - 3 minutes; 36 seconds - deceleration and stop. After the centrifugation process, the blood was separated into four phases. The superior phase comprised serum (blood plasma without fibrinogen and coagulation factors, platelet-poor plasma); the interim phase was a large and dense fibrin block containing CGF; the third phase consisted of white blood cells (WBCs) and stem cells, and the fourth (lower) phase comprised red blood cells (RBCs). CGF (interim phase and third phase) was then separated from the underlying RBC layer (lower phase) with sterile scissors and squeezed manually with wet gauze or using a CGF box to form a CGF membrane, as shown in Figures 1 and 2.

###  Surgical procedure

 After extra- and intra-oral asepsis, the surgical site was anesthetized with 2% lignocaine with adrenaline (1:80 000) using a block or infiltration technique. Then, using Bard Parker blade #15, the sulcular incisions were extended to one tooth on either side of the defect on the facial and lingual/palatal surfaces. A full-thickness mucoperiosteal flap was elevated up to the margins of the alveolar bone using a periosteal elevator. After flap reflection, the soft and hard tissues were subjected to degranulation and debridement using an area-specific Gracey curette, followed by irrigation with 0.9% normal saline solution. The intrabony defect was visualized and assessed after thorough debridement and degranulation.

**Figure 3:**
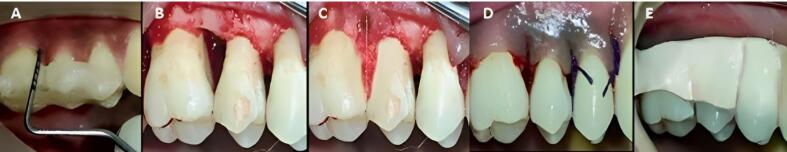
Surgical procedure at experimental site A. A. Preoperative. B. Intrabony defect exposure. C. CGF membrane placement. D. Suture placement. E. Periodontal dressing placement. (CGF: concentrated growth factor)

**Figure 4:**
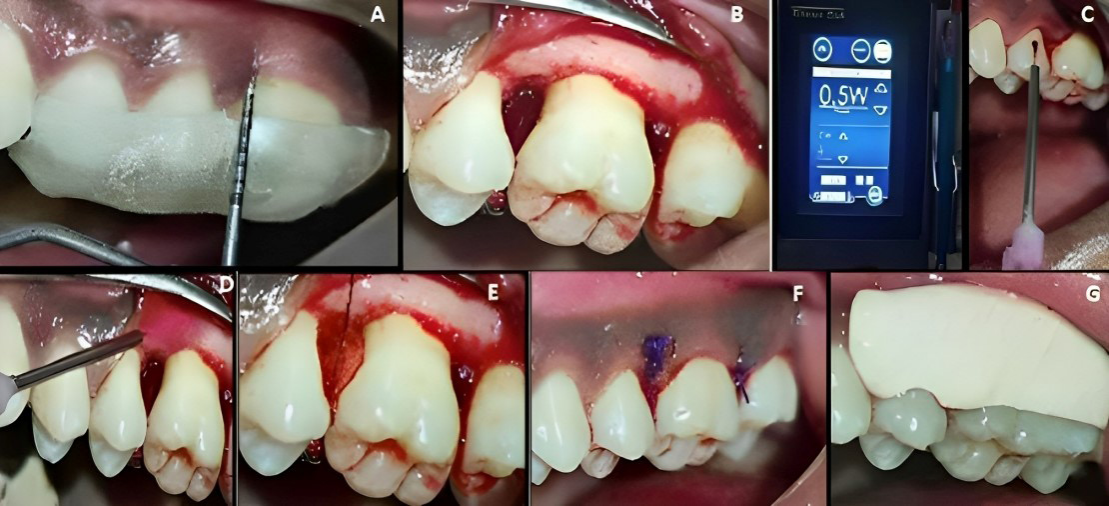
Surgical procedure in experimental site B. A. Preoperative. B. Intrabony defect exposure. C&D. Diode laser application. E. CGF membrane placement. F. Suture placement. G. Periodontal dressing placement. (CGF: concentrated growth factor)

 At experimental site A, after OFD and defect exposure, using 3-0 absorbable sutures, a pre-suturing procedure was carried out before the CGF membrane placement. Then, the defect was covered with a CGF membrane, as shown in Figure 3. At experimental site B, following OFD, the defect area was irradiated with a diode laser of 810 ± 20-nm wavelength at a power output of 1 W in contact, continuous mode through an optic fiber of 400 µm for 30 seconds intermittently (10 seconds/irradiation) in a parallel manner and sweeping motion from coronal to apical part and another application with the same wavelength of the laser used before, but in a non-contact, continuous mode at 0.5 W was used resulting in a complete dose of 4 J/cm^2^/surface on the inner surfaces of the flap and also on the surfaces of the intrabony defect. After laser irradiation, the defect was covered by a CGF membrane, with prior pre-sutures placed, as shown in Figure 4.

 After CGF membrane placement, using a surgeon’s knot, the pre-sutures were tied to achieve a close approximation of the flap at both experimental sites. The periodontal dressing (Coe-Pack TM) was placed. Suitable antibiotics and analgesics were prescribed post-surgically for five days. Instructions to be followed by the patient post-surgically were advised.

###  Recall and evaluation

**Figure 5:**
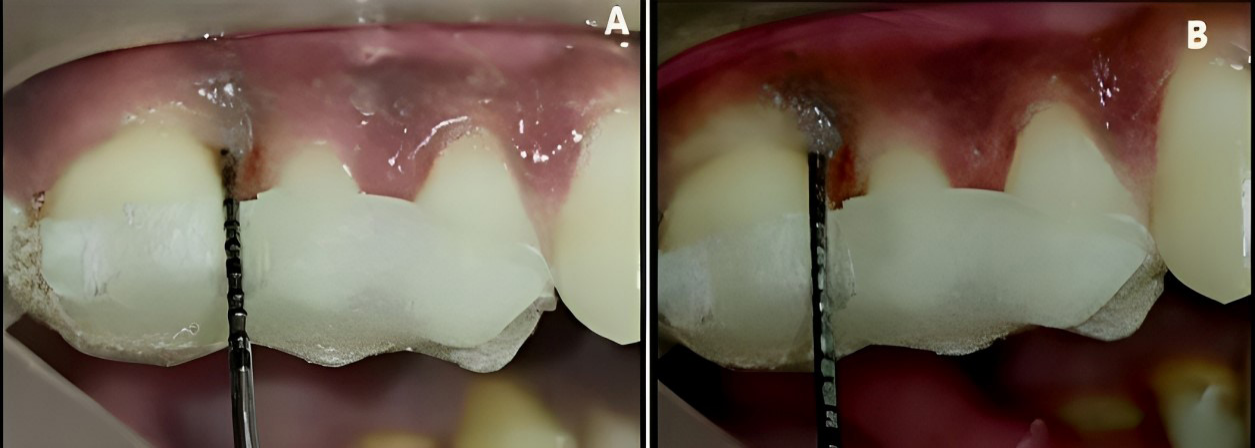
Experimental site A, postoperative-clinical. A. 3 months. B. 6 months

**Figure 6:**
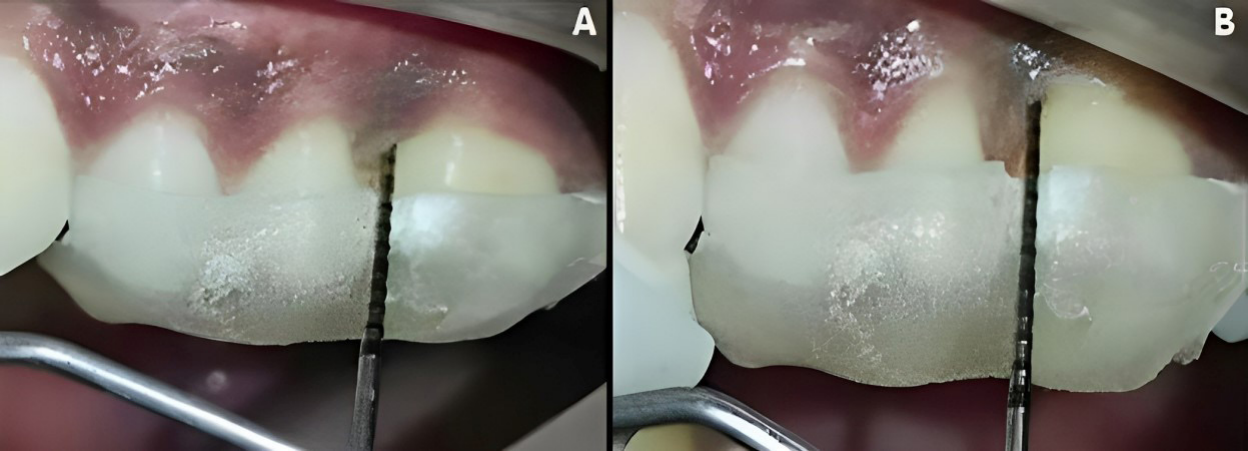
Experimental site B, postoperative-clinical. A. 3 months. B. 6 months

**Figure 7:**
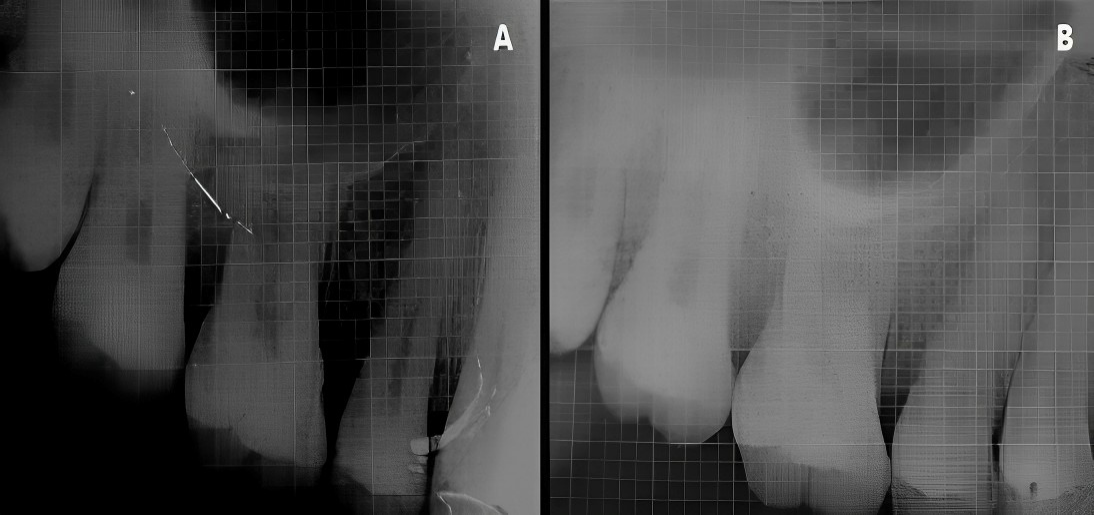
Experimental site A, postoperative-radiographic. A. Baseline. B. 6 months

**Figure 8:**
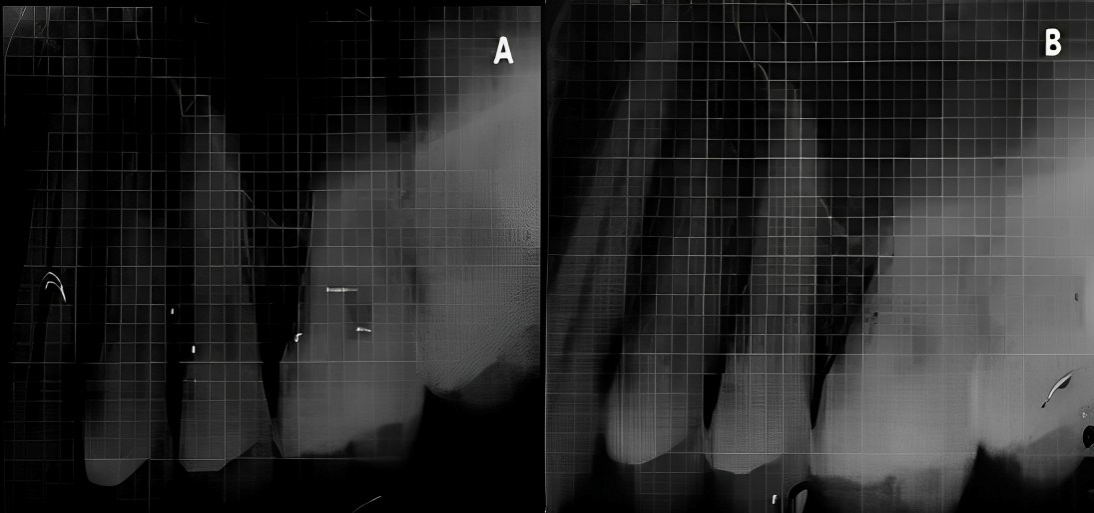
Experimental site B, postoperative-radiologic. A. Baseline; B. 6 months

 Patients were recalled and reviewed at the first, third, and sixth postoperative months, respectively, and correspondingly. All the clinical measurements were recorded at the third and sixth postoperative months, as shown in Figures 5 and 6, and the postoperative radiographs were taken at the end of six months, as shown in Figures 7 and 8.

 Clinical parameters that were evaluated included plaque index (PI), gingival bleeding index (GBI), PPD, and CAL. The evaluated radiographic parameters included bone fill (BF), bone fill percentage (BF%), bone crest change (BCC), and bone crest change percentage (BCC%). The parameters were measured based on the radiographic changes at the base of the defect (BD) and alveolar crest (AC) from the cementoenamel junction (CEJ) and correction factor (CF) as follows.^10,11^

 BF = [CEJ to BD (baseline) – CEJ to BD (post-op)] × CF

 BF% = [BF ÷ CEJ to BD (baseline)] × 100

 BCC = [CEJ to AC (baseline) - CEJ to AC (post-op)] × CF

 BCC% = [BCC ÷ CEJ to AC (baseline)] × 100

**Figure 9:**
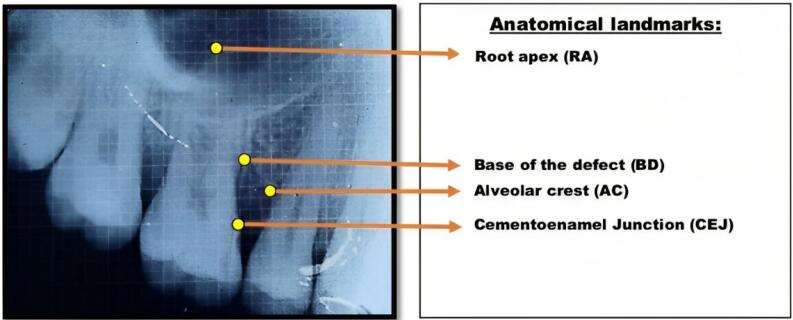
Landmarks to be identified in preoperative and postoperative radiographic images for intrabony defect measurement

 The identification of landmarks in the radiographic images followed the criteria set by Björn et al^12^ and Schei et al^13^ to measure the intrabony defect, as shown in Figure 9. The correction factor (CF) was calculated based on the distance from CEJ to root apex (RA) to estimate distortion between baseline and postoperative radiographs.

 Correction Factor = [CEJ to RA (baseline)] ÷ [CEJ to RA (post-op)]

**Figure 10:**
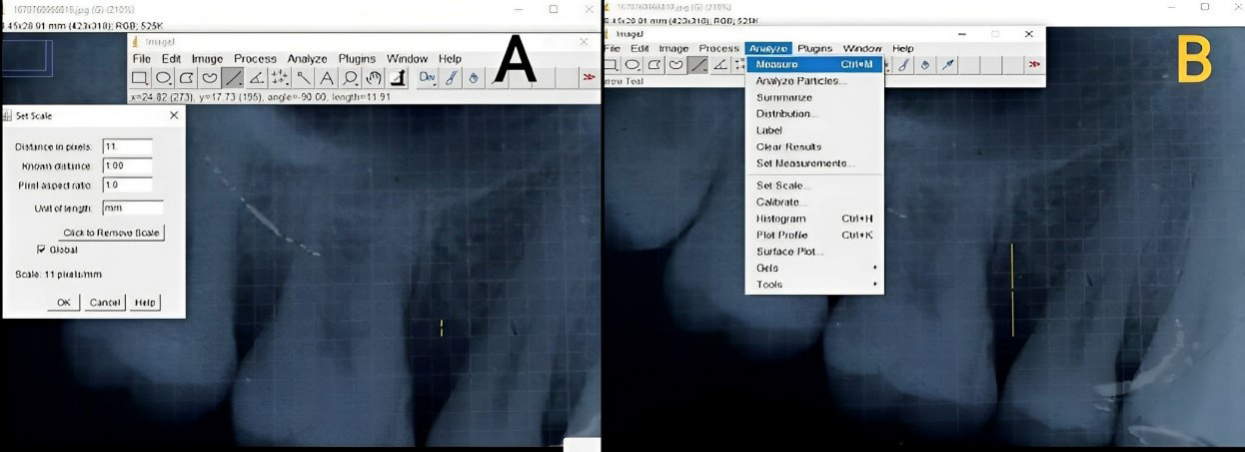
Image J analysis of radiographs. A. Setting a scale of 1 mm per radiographic grid. B. Intrabony defect measurement

 The crown length was measured from the cusp tip to the CEJ in cases where root length measurement was invalid. The linear radiographic parameters were analyzed in millimeters (mm) by Image J software, as shown in Figure 10.

## Results

 Descriptive and inferential statistics were analyzed using SPSS 20.0. Parametric data (PI and GBI) were compared within experimental sites using repeated-measures ANOVA between baseline and three and six months. Non-parametric data (PPD and CAL) were compared within experimental sites using the Friedman test and between experimental sites using the Whitney U test between baseline and three and six months. Non-parametric data (BF, BF%, BCC, and BCC%) were compared between experimental sites using the independent samples t-test at six months, respectively.

###  Clinical parameters

 Concerning plaque and gingival indices, baseline mean plaque and GBI scores were 2.4 ± 0.31 and 75.13 ± 5.95, respectively. A statistically significant reduction was noted at three months (0.89 ± 0.22 and 28.96 ± 3.59) and six months (0.47 and 9.74 and 22.99 ± 3.21) (*P* = 0.000), respectively. Tables 1 and 2 show the mean values of plaque and GBI scores, respectively.

**Table 1:** Comparison of plaque index using repeated measures ANOVA test

**Time interval**	**Plaque index** **(Mean±SD)**	**Mean difference from baseline±SD**	***P*** ** value**
Baseline	2.4 ± 0.31	-	-
3 months	0.89 ± 0.22	1.51 ± 0.09	0.000*
6 months	0.63 ± 0.16	1.77 ± 0.15	0.000*

The data has been represented in mean, mean difference, SD and *P* value. SD: standard deviation; ANOVA: Analysis of Variance. *P values less than 0.05 are considered statistically significant.

**Table 2:** Comparison of Gingival bleeding index using Repeated measures ANOVA test

**Time interval**	**Gingival bleeding index (Mean±SD)**	**Mean difference from baseline±SD**	***P*** ** value**
Baseline	75.13 ± 5.95	-	-
3 months	28.96 ± 3.59	46.17 ± 2.36	0.000*
6 months	22.99 ± 3.21	52.14 ± 2.74	0.000*

SD: standard deviation; ANOVA: analysis of variance. The data has been represented in mean, mean difference, SD and *P* value. *P values less than 0.05 are considered statistically significant.

 Concerning PPD, at baseline, the mean probing depth at experimental sites A and B were 7.30 ± 0.82 mm and 7.30 ± 0.95 mm, which reduced to 3.90 ± 0.74 mm and 3.20 ± 0.79 mm at three months and 3.30 ± 0.48 mm and 2.00 ± 0.67 mm at six months, respectively. Experimental site B showed maximum mean pocket depth reduction of 4.1 ± 0.24 mm and 5.3 ± 0.28 mm at three and six months from baseline compared to the mean pocket depth reduction at experimental site A (3.40 ± 0.085 mm and 4.0 ± 0.34 mm) with a mean difference of 0.70 ± 0.05 mm at three months (*P* = 0.040) and 1.30 ± 0.18 mm at six months (*P* = 0.001), which was highly significant. Tables 3 and 4 show the mean values of PPDs within and between experimental sites, respectively.

**Table 3:** Comparison of probing pocket depth within experimental site A and experimental site B using Friedman test

**Experimental sites**	**Baseline** **Mean±SD (mm)**	**3 months** **Mean±SD (mm)**	**Mean difference baseline -3 months**	**6 months** **Mean±SD (mm)**	**Mean difference baseline – 6 months**	***P*** ** value**
Experimental Site A	7.30 ± 0.823	3.90 ± 0.738	3.40 ± 0.085	3.30 ± 0.483	4.0 ± 0.34	0.000*
Experimental Site B	7.30 ± 0.949	3.20 ± 0.789	4.1 ± 0.24	2.00 ± 0.667	5.3 ± 0.28	0.000*

SD: standard deviation. The data has been represented in mean, mean difference, SD and *P* value. *P values less than 0.05 are considered statistically significant.

**Table 4:** Comparison of probing pocket depth between experimental site A and experimental site B using Mann-Whitney test

**Time interval**	**Experimental site A** **Mean±SD (mm)**	**Experimental site B** **Mean±SD (mm)**	**Mean difference**	***P*** ** value**
Baseline	7.30 ± 0.823	7.30 ± 0.949	0.00	0.967
3months	3.90 ± 0.738	3.20 ± 0.789	0.700 ± 0.05	0.040*
6months	3.30 ± 0.483	2.00 ± 0.667	1.300 ± 0.184	0.001*

SD: standard deviation. The data has been represented in mean, mean difference, SD and *P* value. *P values less than 0.05 are considered statistically significant.

 As for CAL, the mean clinical attachment levels at baseline at experimental sites A and B were 6.80 ± 1.03 mm and 7.20 ± 1.55 mm, which decreased to 3.40 ± 1.08 mm and 3.10 ± 1.29 mm at three months and 2.80 ± 0.79 mm and 1.90 ± 1.10 mm at six months. Experimental site B showed maximum mean clinical attachment gain of 4.10 ± 0.26 mm and 5.3 ± 0.28 mm at three and six months from baseline when compared to the mean clinical attachment gain at experimental site A (3.40 ± 0.042 mm and 4.0 ± 0.25 mm) with a mean difference of 0.30 ± 2.36 mm at three months (*P* = 0.575) and 0.90 ± 1.89 mm at six months (*P* = 0.050), which was statistically significant only at six months. Tables 5 and 6 show the mean values of CALs within and between experimental sites, respectively.

**Table 5:** Comparison of Clinical attachment level within experimental site A and experimental site B using Friedman test

**Experimental sites**	**Baseline** **Mean±SD (mm)**	**3months** **Mean±SD (mm)**	**Mean difference baseline -3months**	**6months** **Mean±SD (mm)**	**Mean difference baseline -6months**	***P*** ** value**
Experimental site A	6.80 ± 1.033	3.40 ± 1.075	3.40 ± 0.042	2.80 ± 0.789	4.0 ± 0.25	0.000*
Experimental site B	7.20 ± 1.549	3.10 ± 1.287	4.10 ± 0.262	1.90 ± 1.101	5.30 ± 0.44	0.000*

SD: standard deviation. The data has been represented in mean, mean difference, SD and *P* value. *P values less than 0.05 are considered statistically significant.

**Table 6:** Comparison of Clinical attachment level between experimental site A and experimental site B using Mann Whitney test

**Time interval**	**Experimental site A** **Mean±SD (mm)**	**Experimental site B** **Mean±SD (mm)**	**Mean difference**	***P*** ** value**
Baseline	6.80 ± 1.033	7.20 ± 1.549	-0.40 ± 0.51	0.558
3months	3.40 ± 1.075	3.10 ± 1.287	0.30 ± 2.36	0.575
6months	2.80 ± 0.789	1.90 ± 1.101	0.90 ± 1.89	0.050*

SD: standard deviation. The data has been represented in mean, mean difference, SD and *P* value. *P values less than 0.05 are considered statistically significant.

###  Radiographic parameters

 The mean bone fill, at six months at experimental sites A and B, was 1.99 ± 0.16 mm and 2.12 ± 0.18 mm, with a mean difference of -0.13 ± 0.02 mm. The mean bone fill percentages at six months at experimental sites A and B were 21.31 ± 2.14 and 23.03 ± 2.17, with a mean difference of -1.71 ± 0.02. The mean BCCs at six months at experimental sites A and B were 0.63 ± 0.09 mm and 0.687 ± 0.090 mm with a mean difference of -0.05 ± 0.002 mm. The mean BCC percentages at six months at experimental sites A and B were 17.932 ± 3.203 and 19.681 ± 3.797 with a mean difference of -1.74 ± 0.594. The bone fill, bone fill percentage, BCC, and BCC percentage were slightly higher at experimental site B than at site A. However, the mean differences were statistically insignificant between the two experimental sites (*P* = 0.089, 0.091, 0.183, 0.280). Table 7 shows the mean values of radiographic parameters between the experimental sites.

**Table 7:** Comparison of radiographic parameters between experimental site A and site B using independent *t* test

**Radiographic parameters**	**Experimental site A** **Mean±SD**	**Experimental site B** **Mean±SD**	**Mean difference**	***P*** ** value**
Bone fill (mm)	1.987 ± 0.155	2.122 ± 0.178	-0.13 ± 0.02	0.089
Bone fill%	21.311 ± 2.143	23.029 ± 2.165	-1.71 ± 0.02	0.091
Bone crest change (mm)	0.632 ± 0.0871	0.687 ± 0.090	-0.05 ± 0.002	0.183
Bone crest change%	17.932 ± 3.203	19.681 ± 3.797	-1.74 ± 0.594	0.280

SD: standard deviation. The data has been represented in mean, mean difference, SD and *P* value. *P values less than 0.05 are considered statistically significant.

## Discussion

 Periodontal regeneration is the restoration of the attachment apparatus in a pathologically exposed root surface through the healing process. Regeneration is a complex biological process that requires locally acting growth factors, intricately regulated cellular interactions, and extracellular matrix components. Periodontal regeneration is a process orchestrated by a series of biological events resulting in cell migration, adhesion, multiplication, and differentiation. As polypeptide growth factors possess potent local factors to regulate major cellular events, their application in periodontal therapy can enhance wound healing and regeneration. The literature reported that placing specific biomaterials/bone grafts improves attachment levels in intrabony periodontal defects more effectively than OFD alone.^14^

 The present study used the CGF membrane as a biomaterial for tissue regeneration. Unlike other platelet concentrates, CGF has better cell separation by the differential continuous rotation process ranging from 2400 to 3000 rpm. It produces fibrin-rich blocks that are larger, denser, and richer in growth factors than other concentrates, thereby showing superior regenerative capacity and higher versatility. Rodella et al^6^ analyzed the tensile strength of various platelet concentrates, concluding that CGF showed higher tensile strength, more growth factors, higher viscosity, and higher adhesive strength than any other platelet concentrate. The growth factor release in platelet-rich plasma was reported to be rapid but not long-term, whereas platelet-rich fibrin (PRF) has a long-term release of growth factors up to 14 days. However, because of denser and larger fibrin blocks with biphasic platelets, CGF has enormous growth factor release and fibrinogen content that contribute to increased osteogenic differentiation and mineralization. Hence, CGF seems to be one of the promising biomaterials that enhance periodontal regeneration by bone healing in a more controlled and effective long-term way.

 The effectiveness of CGF in wound healing in previous studies was better with a lack of adverse reactions, and the healing was considered uneventful in the initial phases, suggesting the use of CGF in periodontal wound healing.^15^

 Laser technology has proved an effective adjunct in periodontal regenerative surgical procedures in various literature, as diode laser is said to have a bactericidal effect that enhances the complete elimination of bacterial toxins, thereby supporting the healing process of periodontal pockets. Diode laser improves the overall health of the periodontium by enhancing clinical parameters and reducing the amount of bacteria present in the periodontal pockets.^16^ Gallium-aluminum-arsenide diode devices promote earlier osteogenesis by the amplified biological reactions stimulating undifferentiated mesenchymal cells into osteoblasts. Low-level laser therapy (LLLT) increases blood circulation to provide a better supply of inorganic salts to the irradiated area, promoting better bone formation.^17^

 The regeneration process was addressed in various aspects by each of the biomaterials discussed. Hence, combinations of one or more techniques have proved promising and may upregulate the regenerative potential than any of the methods used alone. Therefore, the present clinical and radiographic study aimed to evaluate the efficacy of CGF alone and in combinations with diode laser application in managing periodontal intrabony defects.

 Ten patients were selected and included in this study, who were diagnosed with localized stage III grade B/C periodontitis on either side of the same arch with PPD of ≥ 5 mm after phase I therapy and presented with clinical and radiographic evidence of vertical bone loss. Defect morphology plays a dominant role in the healing and regenerative treatment of intrabony defects, as the depth of the intrabony component influences the amount of bone gain and the periodontal attachment. Various literature has documented that clinical attachment improvements are better in deep intrabony defects.^18^ However, few studies reported similar results in shallower defects, as such in deep defects.^19^ Hence, only three-walled and combined defects were selected, as they were correlated positively with periodontal regeneration.^20,21^ The periodontal ligament and the adjacent alveolar bone also increase the vasculature and the cellular contents, thereby enhancing the bridging of defects in three-walled defects.^22^

 The present study adopted a split-mouth design to eliminate and minimize the bias influenced by specific characteristics exhibited by the patient and inter-patient variability, thereby facilitating the interpretation of trials by direct comparison between two experimental sites.^23^ The coin toss method was applied to randomize experimental sites to avoid bias.

 The mean plaque and GBI scores showed statistically significant reductions at three and six months, respectively, and correspondingly. Comparisons between the two experimental sites revealed that the PPD reduction and clinical attachment gain at the third and sixth postoperative months were higher at sites treated with CGF membrane and diode laser than at sites treated with CGF membrane alone. Ebada et al^24^ also reported results similar to the present study, where maximum probing depth reduction from 4.48 ± 0.26 mm to 2.28 ± 0.19 at three months and 1.76 ± 0.12 mm at six months was observed, respectively, at sites treated with PRF and LLLT compared to the sites treated with the PRF system alone. Vaid et al^25^ evaluated the effect of CGF in the treatment of intrabony defect, where a significant pocket depth reduction from 6.40 mm to 4.50 ± 0.97 mm and 3.40 ± 0.70 mm was noted in the third and sixth months, respectively, similar to the present study. Thorat et al^26^ compared autologous PRF with conventional OFD in managing intrabony defects and reported similar results as documented in the present study.

 The bone fill, bone fill percentage, BCC, and BCC percentage were slightly higher in defects treated with CGF and diode laser than at sites treated with CGF alone. However, the differences were statistically insignificant between the two experimental sites. Gamil et al^27^ observed similar results in the management of intrabony defects where the defect depth was reduced from 5.4 ± 0.7 mm to 0.9 ± 0.5 mm at six months postoperatively in defects treated with LLLT and demineralized bone matrix than defects treated with demineralized bone matrix alone with a mean difference of 2.0 ± 0.4 mm, respectively. The results of the present study were similar to the study by Vaid et al,^25^ where the defect area was reduced from 10.55 mm to 6.35 mm at six months postoperatively in the CGF group.Thalaimalai et al^28^ also observed 2.44 mm of radiographic bone fill in patients treated with simplified papilla preservation flap (SPPF) with LLLT + PRF than patients treated with SPPF + PRF alone at the end of six months, similar to the present study in the treatment of intrabony periodontal defects. Petri et al^29^ evaluated the osteoblast-promoting effect of LLLT on human osteoblasts and reported that LLLT stimulated osteoblastic differentiation by modulating the cellular responses and increasing the concentration of alkaline phosphatase, osteocalcin, bone sialoprotein, and bone morphogenic protein. Bereșescu et al^30^ reported new bone formation without inflammatory cells in the defects treated with LLLT compared to those not treated with LLLT in a histologic study.

 In the present study, sites treated with CGF and diode laser application showed better clinical and radiographic outcomes than sites treated with CGF alone. The present study also evaluated the BCCs in the intrabony defects and thus would contribute to assessing the beneficial effects of both CGF and LLLT in bone healing. Surgical re-entry/histologic evaluation is still considered the ‘gold standard’ to evaluate actual periodontal regeneration, which was lacking in the present study due to ethical concerns. The ability to visualize the exact bone levels by periapical radiography is also limited, inherently. Hence, in the future, long-term studies with extensive sampling and advanced clinical, radiologic, and histologic evaluation should be carried out to determine the efficacy of CGF and diode laser application in treating intrabony defects.

## Conclusion

 Both treatment approaches reported successful outcomes in managing intrabony periodontal defects. The synergistic action of CGF and diode laser application has a promising and successful effect on periodontal tissue regeneration by enhancing positive changes in clinical and radiographic parameters. CGF membrane and diode laser application are tolerated well by the periodontal tissues and are clinically effective. Thus, exploring the synergetic action of CGF and diode laser application in periodontal tissue regeneration with advanced technologies and research methodologies would provide a better treatment option to manage intrabony defects.

## Competing Interests

 Authors have no conflict of interest to declare.

## Ethical Approval

 The study was approved by the Institutional Ethical Review Board (4/IERB/2021 on 04.01.2021) of Tamilnadu Government Dental College and Hospital, Tamilnadu Dr. MGR Medical University, Chennai.

## Funding

 None.
